# Protocol for constructing an orthotopic mouse model of metastatic renal cell carcinoma

**DOI:** 10.1016/j.xpro.2026.104444

**Published:** 2026-03-16

**Authors:** Zhihuang Liang, Shufang Chen, Tiezhu Shi, Xiongjun Wang

**Affiliations:** 1 School of Life Sciences, Guangzhou University, Guangzhou, Guangdong 510006, China; 2 Department of Pathology, Changhai Hospital, Naval Medical University, Shanghai 200433, China

**Keywords:** Developmental biology, Cancer, Immunology, Model Organisms

## Abstract

Renal cancer is characterized by an insidious onset and a high tendency for lung metastasis in advanced stages. Here, we present a protocol for constructing an orthotopic mouse model that recapitulates the spontaneous metastasis process of human renal cell carcinoma (RCC). We describe steps for dynamically monitoring tumor growth and metastasis by *in vivo* bioluminescence imaging. We then detail the procedures for performing histopathological analyses. This protocol provides a reliable preclinical platform for investigating RCC metastasis mechanisms and evaluating anti-metastatic therapies.

For complete details on the use and execution of this protocol, please refer to Shi et al.^1^

## Highlights

• Protocol for constructing luciferase-expressing Renca cell lines
• Surgical procedure for implanting Renca-luc cells into the mouse kidney
• Guidance on using *in vivo* bioluminescence imaging for real-time tumor monitoring
• Instructions for observing tumor pathological features via H&E staining

## Abstract (editor highlights)

Publisher’s note: Undertaking any experimental protocol requires adherence to local institutional guidelines for laboratory safety and ethics.

## Graphical abstract

**Figure:**
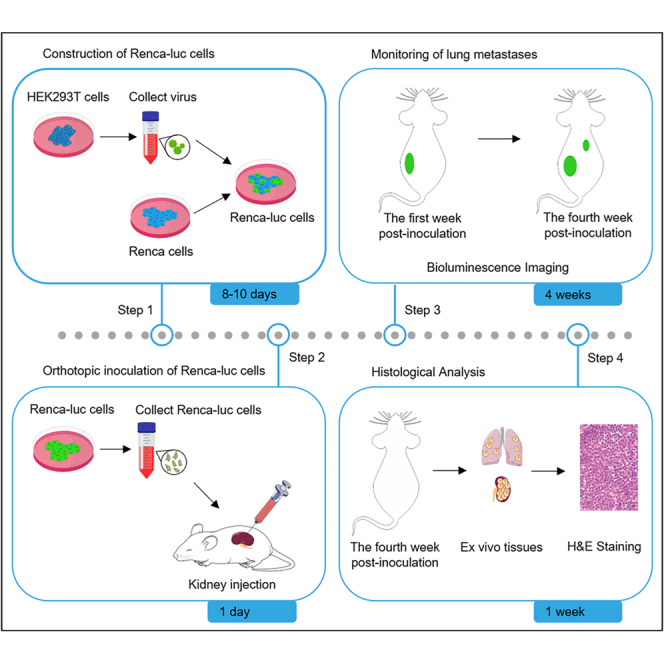

## Before you begin

Metastatic renal cell carcinoma poses a significant clinical challenge due to its poor prognosis and limited treatment options. In this study, we established an orthotopic mouse model to recapitulate the progression of this disease. The model was constructed by subcapsular renal injection of luciferase-labeled murine renal cell carcinoma cells (Renca-Luc) into immunocompetent BALB/c mice. It systematically reproduces the complete pathological process of human metastatic renal cell carcinoma, from local growth to spontaneous metastasis, thereby providing a robust and visualizable preclinical platform for investigating its metastatic mechanisms and evaluating novel anti-metastatic therapeutic strategies.

### Innovation

This protocol introduces a highly reproducible orthotopic mouse model of renal cell carcinoma (RCC) that faithfully recapitulates spontaneous lung metastasis. Its key innovation is the integration of luciferase-labeled tumor cells with a standardized renal subcapsular injection technique in immunocompetent mice, allowing—for the first time—non-invasive, longitudinal, and quantitative tracking of the complete metastatic cascade, from primary tumor development to distant lung colonization, within an intact and physiologically relevant tumor microenvironment. Furthermore, the model combines in vivo bioluminescence imaging with rigorous ex vivo validation through anatomical and histopathological analysis (e.g., H&E staining), providing multi-modal confirmation of both primary and metastatic lesions and significantly enhancing the reliability of experimental outcomes.

### Institutional permissions

All mouse experimental procedures have been accredited by the Association for Assessment and Accreditation of Laboratory Animal Care International and Institutional Animal Care and Use Committee (IACUC). All the mice were maintained and used strictly in accordance with the protocols approved (approval number: 2023-119) by the Research Ethics Committee of Guangzhou University.

### Preparation of target plasmids

**Timing: 1 week**

This section describes the process of obtaining luciferase recombinant plasmids, so that Renca cells can subsequently be transfected to express luciferase.

1. Clone the PCR-amplified firefly luciferase into the pCDH-CMV-MCS-EF1-Puro vector.
2. Transfer the recombinant plasmid into DH5α for amplification.

***Note:*** Transfer conditions: 1 min and 15 s at 42°C, then immediately place on ice for 5 min.

3. Amplify DH5α carrying the recombinant plasmid.

***Note:*** DH5α can be amplified by picking single colonies and shaking them in culture.

4. Extract recombinant plasmids from DH5α.
5. Store at −20°C for subsequent experiments.

## Key resources table

**Table:** 

REAGENT or RESOURCE	SOURCE	IDENTIFIER
**Bacterial and virus strains**		

DH5α Competent cell	Vazyme	C502-02

**Chemicals, peptides, and recombinant proteins**		

D-Luciferin	ThermoFisher	D3571
DMEM medium	Corning	10-013-CVRC
RPMI 1640 medium	Corning	10-040-CVRC
Fetal Bovine Serum (FBS)	Corning	35-079-CV
Phosphate-Buffered Saline (PBS)	Corning	ST066
Trypsin-EDTA (0.25%)	GIBCO	25200072
Penicillin-Streptomycin-Amphotericin B Solution (100×)	Beyotime	C0224-100 mL
Ttypan Blue Stain(0.4%)	GIBCO	2195426
Hematoxylin & Eosin (H&E) Staining Kit	Solarbio	G1120
Polybrene	Beyotime	C0351-1 mL
Puromycin	Solarbio	P8230-25 mg
Isoflurane	RWD	26675-46-7
4% Paraformaldehyde (PFA) Solution	BIOSHARP	BL539A

**Experimental models: Cell lines**		

Renca(wildtype, isolated from the kidney of a male mouse with renal cortical adenocarcinoma)	ATCC	CRL-2947
HEK293T(wildtype, isolated from the kidney of a patient)	ATCC	CRL-3216

**Experimental models: Organisms/strains**		

Mouse:BALB/c(wildtype, male, 5-8weeks)	GemPharmatech	N000020

**Recombinant DNA**		

pCDH-CMV-MCS-EF1-Puro	Addgene	#197989
psPAX2	Addgene	#12260
pMDG.2	Addgene	#12259

**Other**		

Ophthalmic Scissors	SHUANGLU	SL0061
Mouse razor	May Bridge Medical	10144687475673
Needle Holders	JIACHI	10044036898028
Disposable Sterile Syringes	Fenglin Medical	1mL0.45X16RWLB
Disposable Insulin Syringes	Conrad	U40-0.3x8mm
Alcohol Prep Pads	Cofoe	N/A
Ophthalmic Forceps	Shinva	ZO026RB
37°C Warming Pads	PETKIT	100028165256
Surgical Sutures	BeWell Medical	N/A

## Step-by-step method details

### Construction of Renca cells expressing luciferase

**Timing: 8–10 days**

This section describes the method for constructing a Renca cell line expressing luciferase, enabling real-time tracking of tumor progression and metastasis.

**Figure 1:**
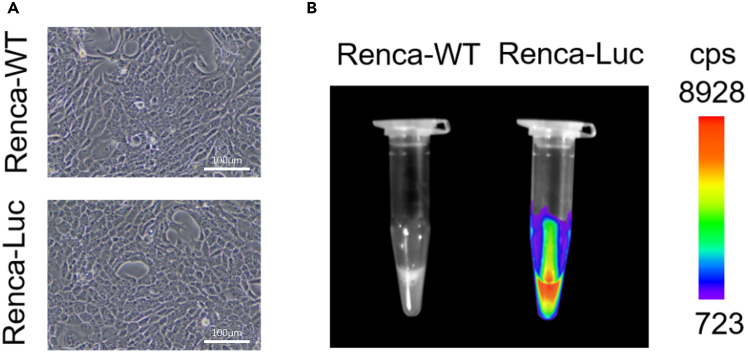
Validation of the luciferase-expressing Renca cell line (Renca-Luc) for in vivo imaging applications (A) Morphological characteristics of Renca-WT (wild-type) and Renca-Luc cells. Scale bar: 100 μm. (B) Bioluminescence imaging conformation of luciferase-expressing Renca cells.
3. Detect luciferase expression.
  a. Collect 1 × 10^6^ Renca-luc cells in a 1.5 mL EP tube.
  b. Add 100 μL of D-luciferin solution (7.5 mg/mL) and mix thoroughly.
  c. Image the sample immediately using the NightOWL II LB 983 in vivo imaging system (Berthold Technologies) (Figure 1B).

***Note:*** Perform parameter setup, image acquisition, and data analysis in accordance with the operating manual of the bioluminescence imaging instrument.

1. Prepare lentiviral solution.
  a. Culture HEK293T cells in 6-cm dishes using DMEM supplemented with 10% Fetal Bovine Serum (FBS) and 100 U/mL Penicillin-Streptomycin.
    **CRITICAL:** Prepare the cell culture medium by combining 44.5 mL of RPMI 1640 medium, 5 mL of fetal bovine serum (FBS; final concentration 10%), and 0.5 mL of Penicillin-Streptomycin-Amphotericin B Solution (100×; final concentration 1%). The solution needs to be thoroughly mixed. This medium can be prepared in advance and stored at −4°C for up to 7 days.
    ***Note:*** Incubate the cells at 37°C in a 5% CO_2_ atmosphere until they reach 60%–70% confluence.
  b. Prepare the transfection mixture by combining 4 μg of plasmid mixture with 16 μL of Polyethylenimine (PEI, 1 μg/μL) in 50 μL of serum-free DMEM.^2^
    ***Note:*** Plasmid mixture composition: psPAX2:pMDG.2:target plasmid = 3:1:4.
  c. Vortex the mixture thoroughly and incubate it at 25°C for 20 min.
  d. Add the mixture to the HEK293T cells.
  e. Replace the medium with fresh DMEM containing 10% FBS and 100 U/mL Penicillin-Streptomycin after 8−12 h of transfection.
  f. Collect the cell supernatant at 48 h and 72 h post-transfection, filter it through a 0.22-μm filter, and pool the filtrate to obtain the lentiviral solution.
    ***Note:*** The lentiviral supernatant may be stored at 4°C for no more than 1 week or at −80°C for no more than 1 year.
2. Infect Renca cells with lentivirus.
  a. Seed Renca cells in 6-cm culture dishes using RPMI-1640 medium supplemented with 10% FBS and 100 U/mL Penicillin-Streptomycin.
    ***Note:*** Incubate the cells at 37°C in a 5% CO_2_ atmosphere.
  b. Passage the cells at a 1:3 ratio into new dishes once they reach 90% confluence with healthy adherent morphology.
  c. Prepare the infection medium by mixing 2 mL of lentiviral solution with 2 mL of RPMI-1640 medium containing FBS and Penicillin-Streptomycin.
  d. Add 5 μL of polybrene solution (1 mg/mL) to the mixture.^3^
  e. Add the prepared infection medium to the cell culture dish and gently swirl the dish to ensure even distribution of the virus.
  f. Remove the virus-containing medium after 12–24 h of infection and replace it with 3 mL of fresh RPMI-1640 medium supplemented with FBS and Penicillin-Streptomycin.
  g. Passage Renca cells at a 1:3 ratio when they reach 90% confluence.
  h. Add 4 μL of puromycin (1 mg/mL) for 48-h selection, while establishing uninfected Renca cells as a negative control (Figure 1A).
    ***Note:*** The control group cells should be completely dead after 48 h of selection.

### Renca-luc cell orthotopic inoculation

#### Surgical instrument preparation

**Timing: 1 day**

Surgical instruments must undergo sterilization to prevent postoperative wound infection in mice.

4. Prepare multiple sets of individually packaged surgical instruments according to the number of mice.

***Note:*** Each set should contain: 1 pair of surgical scissors, 2 pairs of tweezers, 1 needle holder, 2 pieces of gauze, and 10 cotton swabs.

**Figure 2:**
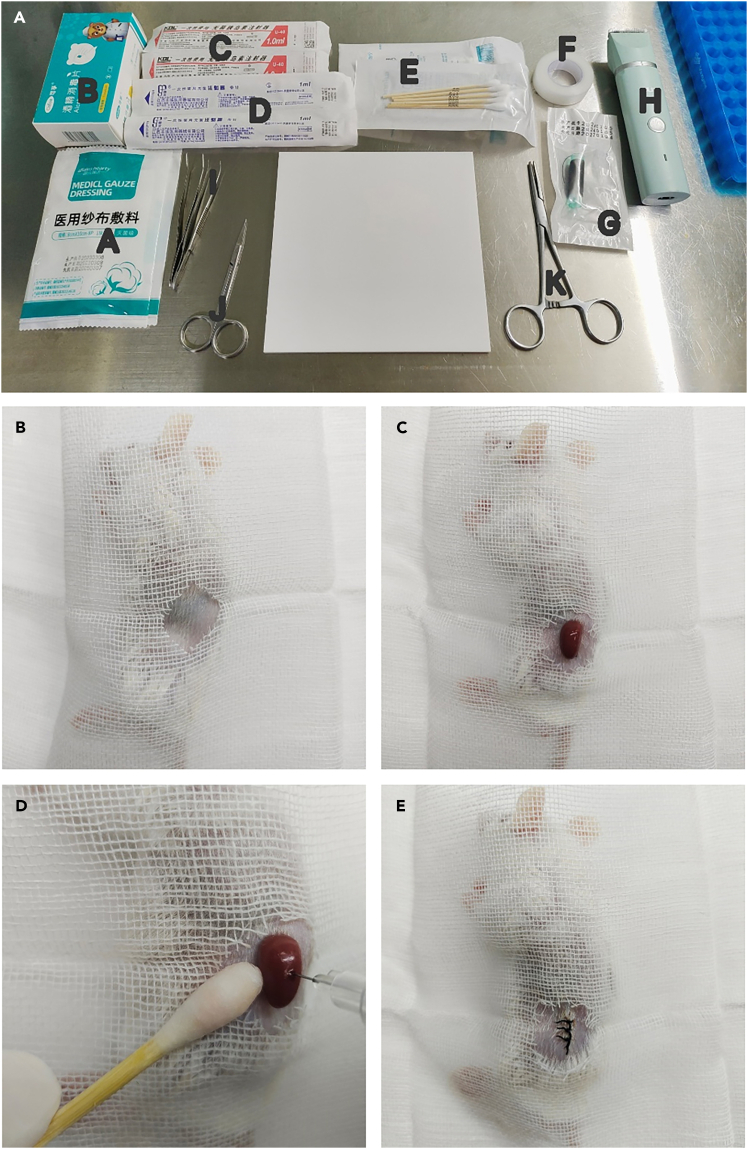
Surgical procedure for orthotopic inoculation of Renca-Luc cells (A) Surgical instruments: A. gauze, B. alcohol swabs, C. insulin syringes, D. syringes, E. cotton swabs, F. adhesive tape, G. sutures, H. hair clippers, I. forceps, J. scissors, K. needle holder. (B) The surgical area was shaved and covered with sterile gauze to control bleeding and prevent contamination. (C) An incision was made below the left costal margin to expose the left kidney. (D) Subcapsular injection of the cell suspension (0.5 × 10^6^ Renca-Luc cells in 25 μL of PBS). (E) Closure of the wound with muscle layer and skin layer, separately.

5. Sterilize the packaged instruments by autoclaving at 121°C for 15 min.
6. Remove the instrument packs and allow them to dry in a clean area for later use (Figure 2A).

#### Preparation of Renca-luc cell suspension

**Timing: 30 min**

This section describes the collection and quantification methods for Renca-luc cells to ensure tumor growth rate remains within a controllable range.

7. Aspirate the culture medium from the dish and wash the cells with PBS.

***Note:*** The day before the surgery, ensure that the confluence of Renca-luc cells in a 10 cm culture dish reaches 60%–70%, and replace with fresh RPMI-1640 medium (containing 10% FBS and 100 U/mL penicillin-streptomycin).

8. Add 1 mL of 0.25% trypsin-EDTA and gently shake the dish to ensure that trypsin fully contacts the adherent cells.
9. Tilt the dish at a 45° angle and gently tap it to loosen the adherent cells.
10. Add 3 mL of RPMI-1640 medium (containing 10% FBS and 100 U/mL penicillin-streptomycin) to stop the digestion.
11. Pipette thoroughly, then transfer the suspension to a 15 mL polypropylene centrifuge tube.
12. Centrifuge at 155 × *g* for 2 min at 4°C, discarding the supernatant.
13. Add 5 mL of PBS to wash the cells, centrifuging under the same conditions as in step 12.
14. Resuspend the cells in 2mL cold PBS.
15. Mix 10 μL of the cell suspension with 10 μL of trypan blue to assess the viability and count of the cells using an automated cell counter.^4^
16. Centrifuge at 155 × *g* for 2 min at 4°C, discarding the supernatant.
17. Resuspend in an appropriate volume of cold PBS to achieve a cell concentration of 2×10^7^ cells/mL.
18. Place the cell suspension in an ice box until ready for use.

***Note:*** The passage number of Renca-luc cells should not exceed 10 generations.

#### Mouse surgical preparation

**Timing: 20 min**

This section describes the anesthesia procedure for mice to ensure adequate anesthesia that does not interfere with the surgical procedure.

19. Turn on the heating pad (37°C) for the warming of the mice’s body temperature after surgery.
20. Prepare multiple mouse cages according to experimental groups placed on the heating pad.
21. Attach the corresponding group labels.
22. Utilize an isoflurane vaporizer to anesthetize the mice.
23. Assess the depth of anesthesia by testing the foot pedal response once the mice enter a sleep state.^5^
24. Use a shaving blade to remove hair from the surgical area.

***Note:*** Select male BALB/c mice aged 5 to 8 weeks from the same batch before surgery, and record the weight of the mice. Mice should be fasted for 6 h before surgery (water is not restricted).

#### Surgical exposure of mouse kidney

**Timing: 10 min**

This section describes the kidney exposure procedure in mice, ensuring the operation is performed while avoiding damage to the organ.

25. Use tape to secure the mouse on the surgical table, exposing the surgical area.
26. Clean the surgical site and surrounding area with alcohol swabs, repeating the cleaning process three times.
27. Place sterile gauze around the surgical site to prevent contamination (Figure 2B).
28. Administer a subcutaneous injection of 100 μL 0.125% bupivacaine for analgesia.
29. Make a 1.5 cm longitudinal incision approximately 1 cm below the left costal margin.

***Note:*** Avoid damaging blood vessels and organs during the incision.

30. Bluntly dissect the muscle layer using scissors and forceps.
31. Gently retract the intestines with a cotton swab to expose the left kidney (Figure 2C).

***Note:*** Perform all surgical procedures within a sterile biological safety cabinet.

#### Tumor inoculation

**Timing: 10 min (for one mouse)**

This section describes the injection of quantified Renca-luc cells into the mouse kidney to ensure consistent initial tumor cell numbers in each mouse.

32. Withdraw 25 μL of Renca-luc cell suspension using a sterile disposable insulin syringe.

***Note:*** Mix the cell suspension using a pipette or vortex mixer before withdrawing the cells.

33. Inject into the renal capsule.
  a. Immobilize the kidney with a PBS-moistened cotton swab.
  b. Insert the needle into the subcapsular space at the lower pole of the kidney to a depth of approximately 2 mm.
  c. Slowly inject the cell suspension.
  d. Withdraw the needle and apply gentle pressure to the injection site with a cotton swab for 1 min to prevent leakage (Figure 2D).

**CRITICAL:** Avoid pushing the needle too quickly to prevent excessive pressure that could lead to capsule rupture.

34. Confirm the absence of leakage or bleeding at the kidney injection site.
35. Lift the muscle layer and reposition the kidney using a cotton swab.
36. Close the inner muscle layer and skin layer respectively using sterile sutures and a needle holder with an interrupted suture technique (Figure 2E).^6^
37. Place the corresponding cage on the electric heating pad, then gently transfer the postoperative mouse into it.

#### Postoperative care and tumor growth monitoring

**Timing: 4–5 weeks**

This section describes the postoperative care for mice and the subsequent monitoring of tumor progression, enabling real-time tracking of tumor development.

38. Maintain awakened mice at 22–26°C under a 12/12 h light/dark cycle, and provide sterilized pellet feed and drinking water ad libitum.
39. Check wound healing status and measure body weight daily for the first three days.

***Note:*** Ensure weight loss does not exceed 10% and monitor overall health condition.

40. Perform bioluminescence imaging 1 week after injection.

***Note:*** Repeat weekly to dynamically monitor tumor progression.

#### Bioluminescence imaging

**Timing: 1–2 h**

This section describes the monitoring of mice using in vivo imaging technology to track the dynamic progression of tumors.

41. Prepare D-luciferin solution.
  a. Prepare a 15 mg/mL stock solution by dissolving D-luciferin potassium salt in sterile phosphate-buffered saline (PBS, pH 7.4).
  b. Vortex the mixture until complete dissolution.
  c. Sterilize the solution by passing it through a 0.22 μm filter.
  d. Aliquot the solution into 1.5 mL microcentrifuge tubes.
  e. Store the aliquots at −80°C for future use.

***Note:*** Protect from light during preparation and storage. Avoid repeated freeze-thaw cycles. Discard unused reagent after single thaw.

42. Inject D-luciferin solution into mice.
  a. Place the mouse in an induction chamber pre-filled with 2-3% isoflurane (oxygen flow rate: 0.8–1 L/min).
  b. Transfer the anesthetized mouse to the imaging platform in a supine position.
  c. Administer the D-luciferin solution via slow intraperitoneal injection.
  d. Apply gentle pressure to the injection site for 10 sec to prevent leakage.
  e. Wait 5−10 min to allow sufficient substrate-enzyme reaction before imaging.

**CRITICAL:** Administer D-luciferin at 150 mg/kg body weight (e.g., for a 25 g mouse: dose = 3.75 mg, volume = 250 μL).^7^

43. Perform imaging using the NightOWL II LB 983 system.

#### Organ harvesting

**Timing: 30 min**

This section describes the collection procedure of tumor-bearing organs for observing tumor development.

44. Euthanize the mice.
  a. Induce anesthesia with 5% isoflurane inhalation.
  b. Maintain anesthetic depth, then perform euthanasia via cervical dislocation.

**CRITICAL:** Avoid chemical euthanasia agents (e.g., CO_2_) that may confound subsequent metabolic analyses.

45. Expose the mouse’s chest.
  a. Secure the mouse in a supine position on a dissection board.
  b. Disinfect the thoracic and abdominal skin with 75% ethanol.
  c. Make a midline skin incision along the line from the xiphoid process to the mandibular symphysis.
  d. Separate the subcutaneous tissue to expose the sternum.
  e. Cut through the costal cartilage along the sternal midline using micro-scissors.
    ***Note:*** Avoid damaging lung tissue.
  f. Retract the anterior chest wall to fully expose the thoracic cavity.
46. Perfuse the lung.
  a. Insert a 23-gauge blunt needle into the left ventricle.
  b. Perfuse with 10 mL of pre-chilled PBS at a flow rate of 3 mL/min.
  c. Continue perfusion until the lung tissue turns pale.

***Note:*** Create an outflow tract by incising the right atrium to ensure complete perfusion. This step clears red blood cells to reduce background fluorescence.

47. Harvest lung.
  a. Grasp the upper trachea with micro-dissection forceps.
  b. Dissect the mediastinal connective tissue along the tracheoesophageal groove.
  c. Transect the trachea at the tracheoesophageal junction.
  d. Separate tissues caudally toward the diaphragm to mobilize the lung tissue.
  e. Remove surrounding adipose tissue and lymph nodes from the lung.
  f. Harvest the intact heart-lung bloc en bloc.
48. Harvest Kidney.
  a. Grasp the adipose or connective tissue at the superior pole of the kidney for traction.
  b. Dissect the surrounding adipose tissue, and renal blood vessels using fine scissors or forceps.
  c. Excise the target kidney intact.
49. Fix the tissue.
  a. Rinse the lung tissue with ice-cold PBS to remove residual blood.
  b. Immerse the tissue in 4% paraformaldehyde solution.
  c. Fix the samples at 4°C for 24–48 h.

***Note:*** Avoid exceeding 72 h of fixation as this may increase tissue brittleness.^8^

#### H&E staining and histopathological analysis

**Timing: 1 week**

This section describes the HE staining procedure for tumor-bearing organs to allow direct visualization of tumor morphology and size.

50. Embed the tissue in paraffin.
  a. Dehydrate the tissue.
    ***Note:*** Perform gradient dehydration using an automated tissue processor: 70% ethanol (60 min) → 80% ethanol (60 min) → 95% ethanol (60 min) → 100% ethanol I (45 min) → 100% ethanol II (45 min) → xylene I (30 min) → xylene II (30 min).
    **CRITICAL:** Ensure anhydrous conditions during 100% ethanol steps, as any water residue will compromise clearing efficiency.
  b. Infiltrate and embed the tissue in paraffin.
    i. Transfer the dehydrated lung tissue to molten paraffin at 65°C and perform two 90-min immersions.
    ii. Embed the tissue using a pre-warmed metal mold in the embedding station.
    iii. Orient the tissue in the mold according to its anatomical position.
    iv. Fill the mold with 60°C paraffin to completely cover the tissue.
    v. Solidify the block by transferring the mold to a cold plate.
      ***Note:*** Initiate paraffin melting 3–4 h prior to removing tissue from the processor to ensure paraffin is fully molten at embedding.
51. Cut the tissue paraffin blocks into slices.
  a. Trim the paraffin block to expose target regions.
  b. Cut 5 μm-thick serial sections using a rotary microtome.
  c. Float sections on a 40°C deionized water bath for flattening.
  d. Mount onto charged slides and tilt-drain excess water.
  e. Dry slides in a 37°C oven for 12 h to ensure firm section adhesion.
52. Perform H&E staining on tissue sections.
  a. Dissolve tissue sections in paraffin.
    i. Bake dried slides at 65°C for 20 min.
    ii. Vertically immerse in xylene I for 10 min.
    iii. Transfer to xylene II for 10 min to dissolve paraffin completely.
  b. Perform gradient rehydration.
    **CRITICAL:** Sequentially immerse slides in: 100% ethanol (3 min) → 95% ethanol (3 min) → 85% ethanol (3 min) → 75% ethanol (3 min) → distilled water (2 min).
    ***Note:*** Ensure complete submersion at each step.
  c. Perform hematoxylin staining.
    i. Stain tissue sections in hematoxylin solution for 2 min at 25°C.
    ii. Monitor staining under microscopy until optimal contrast is achieved.
      ***Note:*** Nuclei: deep blue; cytoplasm: light blue.
    iii. Rinse off non-specific staining.
    iv. Store sections in distilled water.
  d. Perform eosin Staining.
    i. Stain the sections in eosin solution for 20 s.
      ***Note:*** The staining time can be adjusted as needed from 15 to 60 s.
    ii. Monitor until the cytoplasm develops a distinct pink coloration.
    iii. Rinse twice with distilled water for 10 s each to remove unbound dye.
  e. Perform gradient dehydration and clearing.
    **CRITICAL:** Quick dips: 75% ethanol (5 dips) → 85% ethanol (5 dips) → 95% ethanol (5 dips) → 100% ethanol (60 s) → xylene (2 min).
  f. Mount with Neutral Resin.
    i. Remove slides from xylene.
    ii. Blot edge residuals.
    iii. Apply DPX Mountant.
    iv. Lower a coverslip at an angle to avoid bubbles.
    v. Cure at 25°C for 24 h.
53. Examine the tissue sections using an optical microscope.

## Expected outcomes

This study successfully established an orthotopic model mimicking the pathological progression of human metastatic RCC in immunocompetent BALB/c mice through renal subcapsular injection of luciferase-labeled murine renal cell carcinoma cells (Renca-Luc). At 4 weeks post-operation, in vivo bioluminescence imaging and histopathological analysis confirmed the formation of primary renal tumors and spontaneous pulmonary metastases.

**Figure 3:**
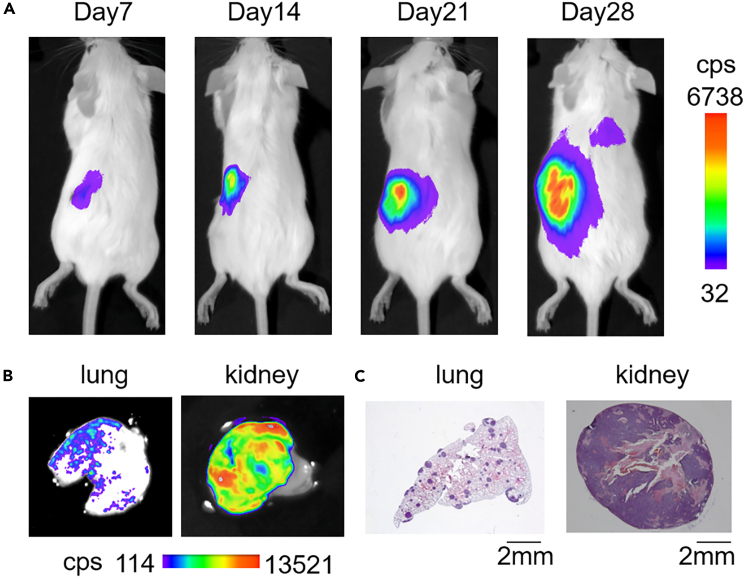
Longitudinal monitoring of renal cell carcinoma progression and spontaneous lung metastasis (A) Bioluminescence imaging of mice at indicated time points after orthotropic implantation of Renca-luc cells. (B) Ex vivo imaging of the lung and kidney harvested on postoperative day 28. (C) Histopathological validation of lung metastatic foci (left) and primary kidney tumor (right) by H&E staining. Scale bar: 2 mm.

Bioluminescence imaging results demonstrated detectable bioluminescence signals in the renal region as early as day 7 post-operation, with signal intensity progressively increasing over time in the primary site and lung, indicating tumor progression and lung metastases formation (Figure 3A). By day 28, ex vivo imaging further verified the presence of primary renal tumors and pulmonary metastatic foci (Figure 3B). Histological analysis further confirmed renal tumor and lung metastases structures present (Figure 3C).

The core advantage of this model lies in its ability to recapitulate the natural progression of RCC from local growth to distant metastasis, enabling non-invasive, dynamic, and quantitative monitoring through Bioluminescence imaging. This provides a highly visualizable and reproducible preclinical platform for investigating RCC metastasis mechanisms and evaluating anti-metastatic therapeutic strategies.^1^ Furthermore, the use of immunocompetent mice preserves intact tumor-immune microenvironment interactions, making it particularly suitable for studying immunomodulation and combination therapies.

In conclusion, the established orthotopic RCC model demonstrates robust metastatic phenotype and high reproducibility, making it suitable for exploring RCC metastasis mechanisms and evaluating drug efficacy. Its significance is underscored by the current scarcity of spontaneous metastasis models. Future studies may further optimize the model by incorporating humanized tumor cells or genetic editing techniques to enhance its clinical translational potential.

## Limitations

This protocol describes the establishment of an orthotopic mouse model of RCC designed to study spontaneous lung metastasis with high reproducibility and surgical precision. Compared to other methods such as subcutaneous implantation or tail vein injection, this model recapitulates key stages of human RCC progression—including primary tumor growth, local invasion, and distant lung metastasis—within a physiologically relevant tissue microenvironment. The major advantage lies in its ability to mirror the natural history of kidney cancer metastasis, thereby providing a robust platform for investigating molecular mechanisms, biomarker discovery, and therapeutic evaluation. However, the model has several limitations: first, the surgical procedure is technically demanding and requires specialized microsurgical skills to ensure consistency and avoid leakage of tumor cells into the peritoneal cavity; second, the incidence and kinetics of metastasis are highly dependent on the choice of cell line (e.g., Renca, RAG, or 786-O) and host strain (e.g., immunocompetent vs. immunodeficient mice), which may affect experimental reproducibility; third, longitudinal monitoring of metastatic spread often requires advanced in vivo imaging modalities, increasing the cost and complexity of studies; fourth, the model may not fully capture the heterogeneity of human RCC, particularly in terms of genetic diversity and tumor-stroma interactions; and fifth, variability in postoperative recovery and immune responses can introduce additional confounding factors. Despite these challenges, the model remains a valuable tool for preclinical research aimed at understanding RCC metastasis and developing novel treatments.

## Troubleshooting

### Problem 1

No bioluminescent signal detected from Renca-luc cells in vitro, related to steps 1–3.

### Potential solution

Verify the success of puromycin selection and ensure the negative control cells are completely dead. Use fresh, aliquoted D-luciferin potassium salt solution protected from light.

### Problem 2

Renal capsule rupture during orthotopic injection, related to steps 33–38.

### Potential solution

Strictly control the needle insertion depth to 2 mm. Inject the cell suspension slowly and steadily. Ensure the insulin syringe needle is sharp and sterile.

### Problem 3

High postoperative mortality of mice, related to steps 39–41.

### Potential solution

Ensure all surgical instruments are sterile and procedures are performed aseptically. Maintain mice on a heating pad until fully recovered from anesthesia.

### Problem 4

No metastatic lesions were found in the lung during endpoint analysis, related to steps 50–53.

### Potential solution

Extend the experimental period to 5–6 weeks to allow more time for metastasis development.

### Problem 5

Excessive background fluorescence or poor morphology in lung tissue sections, related to steps 50–53.

### Potential solution

Ensure the perfusion is adequate until the lung turn completely white. Fix tissues in 4% PFA for 24–48 h (not exceeding 72 h). Strictly follow the dehydration and transparency procedures, ensuring the ethanol and xylene are replaced regularly to avoid moisture absorption.

## Resource availability

### Lead contact

Further information and requests for resources and reagents should be directed to and will be fulfilled by the lead contact, Xiongjun Wang (wangxiongjun@gzhu.edu.cn).

### Technical contact

For technical specifics on executing the protocol, Tiezhu Shi (shitiezhu1991@163.com) will provide support to ensure its correct implementation.

### Materials availability

This study did not generate new materials.

### Data and code availability

This study did not generate datasets.

## Acknowledgments

This research was supported by the 10.13039/501100002858China Postdoctoral Science Foundation (2024M750624) and the 10.13039/501100001809National Natural Science Foundation of China (82403492).

## Author contributions

Z.L., S.C., and T.S. set up and performed the experiments. Z.L., T.S., and X.W. wrote the manuscript.

## Declaration of interests

The authors declare no competing interests.
